# Relationship between the severity of agitation and quality of life in residents with dementia living in German nursing homes - a secondary data analysis

**DOI:** 10.1186/s12888-021-03167-5

**Published:** 2021-04-13

**Authors:** Kathrin Schmüdderich, Daniela Holle, Armin Ströbel, Bernhard Holle, Rebecca Palm

**Affiliations:** 1 German Center for Neurodegenerative Diseases (DZNE), Stockumer Str. 12, Witten, 58453 Germany; 2 Witten/Herdecke University, Faculty of Health, School of Nursing Science, Stockumer Str. 12, Witten, 58453 Germany; 3 University of Applied Sciences (hsg Bochum), Department of Nursing Science, Gesundheitscampus 6-8, Bochum, 44801 Germany; 4 Universitätsklinikum Erlangen, Center for Clinical Studies, Krankenhausstraße 12, Erlangen, 91054 Germany

**Keywords:** Agitation, Aggression, Quality of life, Dementia, Nursing home

## Abstract

**Background:**

Severe agitation and its relation to single dimensions of quality of life are not well understood. The aim of this study was to gain more knowledge about severe agitation and to examine the relationships between the severity of agitation and single dimensions of quality of life among residents with dementia living in German nursing homes.

**Methods:**

This exploratory secondary analysis included data from 1947 residents of 66 German nursing homes from the DemenzMonitor study. The construct of agitation was defined as a composite score of the items *agitation/aggression, irritability/lability* and *disinhibition* from the Neuropsychiatric Inventory Questionnaire (NPI-Q); the resident was classified as severely agitated if at least one of these symptoms was rated as ‘severe’. The single dimensions of quality of life were measured with the short version of the QUALIDEM instrument. To avoid selection bias, two controls with mild or no agitation were selected for each resident with severe agitation using propensity score matching. Mixed linear regression models were then generated to determine the differences in the dimensions of quality of life for the severity of agitation and the defining items.

**Results:**

For four out of five dimensions of quality of life of the short version of QUALIDEM, residents with severe agitation had significantly lower values than residents without severe agitation. Converted to scale size, the greatest difference between both groups was found in the dimension *social isolation* with 23.0% (-2.07 (95% CI: -2.57, -1.57)). Further differences were found in the dimensions *restless tense behaviour* with 16.9% (-1.52 (95% CI: -2.04, -1.00)), *positive affect* with 14.0% (-1.68 (95% CI: -2.28, -1.09)) and *social relations* with 12.4% (-1.12 (95% CI: -1.54, -0.71)).

**Conclusions:**

Severe agitation is a relevant phenomenon among nursing home residents with dementia and is associated with lower values of quality of life in the dimensions *social isolation, restless tense behaviour, positive affect* and *social relations* from the QUALIDEM instrument. Therefore, more attention should be paid to severe agitation in nursing practice and research. Moreover, care strategies used to reduce severe agitation should be considered in terms of their impact on the dimensions of quality of life.

**Supplementary Information:**

The online version contains supplementary material available at (10.1186/s12888-021-03167-5).

## Background

Approximately 50 million people worldwide suffer from dementia, and this number is expected to rise to 152 million by 2050 [1]. In Germany, more than 1.6 million people live with dementia. According to statistical forecasts, the number of people with dementia in Germany will rise to 2.8 million by 2050 [2]. In addition to cognitive impairment, neuropsychiatric symptoms are the main features of dementia. During the course of their disease, approximately 90% of people with dementia develop at least one clinically significant neuropsychiatric symptom [3, 4]. One of the most common neuropsychiatric symptoms experienced by nursing home residents with dementia is agitation [5]. The prevalence of agitated behaviour among nursing home residents with dementia can reach up to 82% depending on the assessment tool used [5, 6]. Regarding the course of neuropsychiatric symptoms in nursing homes, a longitudinal study carried out over 53 months showed that agitation, disinhibition, irritability and apathy have the highest persistence with more than 50% each [7]. Recent studies also investigated the intensity of agitation in terms of its frequency or severity. The two-week prevalence of very frequent agitation in nursing homes is 7.4% according to a study from the Netherlands [8]. With 6.3%, a study in German nursing homes showed comparable results for the prevalence of severe agitation [9].

Various terms are used to describe and define agitation. These include aggression, hyperactivity or irritability. However, definitions differ on whether agitation includes aggression or aggression should be considered separately [10]. According to the clinical and scientific definition from the expert group of the International Psychogeriatric Association (IPA) and the definition from Cohen-Mansfield and her colleagues, aggression is understood as a part of agitation [11, 12]. The IPA defines agitation as 1) ’manifesting excessive motor activity, verbal aggression or physical aggression’ that 2) is associated with emotional stress for the person concerned, 3) occurs in people with cognitive impairment or dementia syndrome, and 4) ’is not solely attributable to another disorder’ [12]. Cohen-Mansfield and her colleagues define agitation as ’inappropriate verbal, vocal or motor activity that is not explained by needs or confusion per se’ [13]. They therefore understand it as behaviour that is considered inappropriate by others and divide it into three syndromes: aggressive behaviour (physical or verbal), physical non-aggressive behaviour, and verbal non-aggressive behaviour [11].

The consequences of agitation are similar to those of neuropsychiatric symptoms in general. For the person with dementia, agitation is associated with functional dependence, higher care costs and early institutionalisation [14]. For relatives and formal caregivers, agitation is one of the most distressing and psychologically challenging neuropsychiatric symptoms [15, 16]. This stress and strain increase with the severity of agitation [17]. Nurses who are frequently exposed to agitated behaviour also report a reduced state of health, reduced ability to work and increased burnout rates [18].

The quality of life of people with dementia is currently not defined consistently. However, it is recognised that the quality of life of people with dementia is subjective and relates to well-being in several dimensions of life [19–21]. As dementia cannot be cured, maintaining and promoting quality of life is one of the most important goals in clinical practice and health care research for people with this disease. For this reason, quality of life has become an important outcome parameter in intervention studies, especially in psychosocial interventions, in this population [22–24].

Previous studies showed a correlation between agitation and the total score of quality of life [25–36]. This correlation is independent of whether agitation was defined using the Cohen-Mansfield Agitation Inventory (CMAI) [27, 29, 31], the Neuropsychiatric Inventory Questionnaire (NPI) [25, 26, 30, 32–36], or a self-developed symptom description [28]. Moreover, both the construct of agitation [25, 32, 34, 35], which was defined by the NPI items *agitation/aggression, irritability/lability* and *disinhibition* (and *euphoria*), and the individual items *agitation/aggression, irritability/lability* and *disinhibition* were associated with the total score of quality of life of people with dementia [26, 30, 33]. Only Woods et al. (2014) could not identify an association between the NPI item *disinhibition* and the total score of quality of life [36]. In addition to agitation, the following variables were most frequently associated with quality of life in these studies: cognitive and functional ability, NPI total score, apathy, depression, eating and nighttime behaviours, anxiety, delusions, hallucinations, medication use, age, sex and presence of pain.

Although Livingston et al. (2014) assumed that agitation is not associated with every dimension of quality of life and that more knowledge is needed about its relationships to single dimensions [37], only Gräske et al. (2014), Henskens et al. (2019), Mjørud et al. (2014) and van Kooten et al. (2017) investigated the dimensions of quality of life in relation to agitation [27, 29, 32, 34]. In addition, none of the studies addressed the severity of agitation. Palm et al. (2018) and Veldwijk-Rouwenhorst et al. (2017) showed that residents with dementia with severe agitation or with very frequent agitation differ from residents with dementia without agitation or with less frequent agitation in terms of sociodemographic characteristics, severity of dementia and neuropsychiatric symptoms [8, 9]. For this reason, we assumed that they might also differ in the dimensions of their quality of life. Since knowledge about severe agitation is very limited, the aim of this study was therefore to gain a better understanding of severe agitation and to examine the relationships between the severity of agitation and single dimensions of quality of life.

## Methods

### Study design

This study represents a secondary data analysis and is based on an exploratory approach as there is no theoretical or empirical evidence of the relationships that can be proven. All methods were performed in accordance with the guidelines and recommendations for secondary data analysis [38].

The data were derived from the DemenzMonitor study (2012-2014), a prospective, observational study [39]. For the DemenzMonitor study, a convenience sample of 66 German nursing homes with 140 care units was recruited through public announcements in newsletters and magazines and at national conferences. As an open cohort, the sample consists not only of residents recruited primarily in 2012 who were observed prospectively but also of care units and residents who were newly included in the sample in 2013 and 2014. Participation in the study was voluntary, and the individual nursing homes decided in how many care units data could be collected and how long they wanted to participate in the study. Those residents who had given their informed consent or whose legal representatives had given their informed consent were included in the study [39, 40].

The secondary data analysis included participants who had been medically diagnosed with dementia and had at least mild cognitive impairment according to the Dementia Screening Scale (DSS) [41], which is outlined later in the manuscript. Since dementia diagnoses in Germany are partly inadequate, this double condition ensured that only residents who truly had dementia were included [42]. The three datasets of the measurements from 2012, 2013, and 2014 were pooled. To avoid bias caused by including residents more than once, only the first measurement from each resident was included in the analysis.

### Data collection

Data from the DemenzMonitor study were collected by the nursing staff once a year over a period of one month. One staff member of each nursing home (study coordinator) was trained in the data collection procedures via a one-day lecture and was responsible for ensuring that the data collection guidelines were followed. The study coordinator either assessed the residents him- or herself or trained other nurses. It was intended that the assessments of the residents should always be carried out by the nurse who was most familiar with the resident. The data were documented by paper and pencil or by directly entering the data into an online database [39].

### Measurements

The residents’ sociodemographic data were obtained from medical records; these included age, sex, length of stay in months, the existence of a court order to stay in the nursing home and the number of visits. The quality of life, cognitive and functional abilities and neuropsychiatric symptoms of the residents were rated with proxy assessment tools.

For the measurement of quality of life, the German version of the QUALIDEM instrument was used, which was originally developed in the Netherlands specifically for residents with dementia. The instrument allows a retrospective proxy assessment of quality of life and can be administered to people with mild (long version) to very severe (short version) dementia [43]. In the short version for people with severe dementia, the instrument we used here, quality of life is operationalised in six dimensions (also called subscales) with a total of 18 items: *care relationship* (3 items), *positive affect* (4 items), *negative affect* (2 items), *restless tense behaviour* (3 items), *social relations* (3 items), and *social isolation* (3 items). Each item is assessed with four possible answers (never, rarely, sometimes, frequently); higher scores indicate a better quality of life in the respective dimension [44]. The German short version of the QUALIDEM instrument shows moderate to high internal consistency, strong intra-rater reliability and good feasibility [45, 46]. To achieve strong inter-rater reliability, quality of life was assessed by more than one person according to the recommendations [45].

Neuropsychiatric symptoms were assessed with the Neuropsychiatric Inventory Questionnaire (NPI-Q), a retrospective questionnaire that measures the presence of a number of neuropsychiatric symptoms (0=present; 1=not present) and their severity (1=mild; 2=moderate; 3=severe). It includes the items *delusions*, *hallucinations*, *depression/dysphoria*, *anxiety*, *apathy/indifference*, *disinhibition*, *irritability/lability*, *agitation/aggression*, *aberrant motor*, *nighttime disturbances* and *eating disturbances*. The NPI-Q is considered reliable and valid [47].

Cognitive impairment of the residents was measured with the Dementia Screening Scale (DSS), a seven-item proxy rating scale. Higher scores indicate a stronger cognitive impairment (range 0-14) [41]. We applied the recommended cut-off score to identify participants with cognitive impairment (DSS score >2) [41]. The Physical Self-Maintenance Scale (PSMS) was used to assess physical functions and self-care abilities. Higher scores indicate a stronger impairment of functional ability (range 6-30) [48].

### Variables

We assessed the dimensions *positive affect*, *negative affect*, *restless tense behaviour*, *social relations* and *social isolation* of the short version of the QUALIDEM instrument to quantify quality of life. Since the items of the dimension *care relationship* of the QUALIDEM instrument overlapped with the questions on the construct of agitation, this dimension was not investigated.

To assess agitation, we defined the construct of agitation as a composite score of the NPI-Q items *agitation/aggression*, *disinhibition* and *irritability/lability*, based on a previous mokken analysis of the DemenzMonitor dataset [9] and the results of other studies [49, 50]. This definition provided a measurable conceptualization of agitation that included both verbal and physical aggressive behaviours and verbal and physical non-aggressive behaviours, such as refusing help or exhibiting irritability and impatience. To compare residents with severe agitation with residents without severe agitation, we established three agitation categories: 1) severe agitation: a score of 3 in at least one of the three NPI-Q items (*agitation/aggression*, *disinhibition*, or *irritability/lability*); 2) no agitation: a score of zero in all three agitation NPI-Q items; and 3) mild agitation: all scores between these two categories. For the analyses, residents with mild or no agitation were grouped together.

We identified age, sex, DSS score, visit, length of stay in months, PSMS score and the other NPI-Q items as possible confounders on a theoretical basis [19, 51]. Since the items *feeding*, *dressing*, *grooming* and *bathing* of the PSMS ask for resistance behaviour [48], they were excluded because of their similarity to the descriptions of agitation (e.g., PSMS *feeding*: ‘Does not feed self at all and resists efforts of others to feed him/her.’ versus NPI-Q *agitation/aggression*: ‘Is the patient resistive to help from others at times, or hard to handle?’). The PSMS items *toileting* and *physical ambulation* were retained as possible confounders. With respect to the NPI-Q items, it was difficult to classify them into the theoretical understanding of confounders, mediators, and colliders. Therefore, all other NPI-Q items were initially considered as possible confounders.

### Data analysis

We calculated relative and absolute frequencies or means and standard deviations to describe sociodemographic, functional, cognitive, and neuropsychiatric characteristics.

Before we analysed the differences between the two groups, we used a matching method according to the steps outlined by Ho and colleagues to avoid bias due to unequal distributions of characteristics [52, 53]. For each resident with severe agitation, the matching procedure attempted to find one or more residents with mild/no agitation with comparable characteristics in the matching variables, simulating a random assignment of residents to agitation stages. As matching variables, we selected the possible confounders [54]. For the matching procedure, we chose an individual 1:2 matching along the nearest neighbour based on the propensity score (PS) [52, 55]. The PS describes ’the conditional probability of assignment to a particular treatment given a vector of observed covariates’ [56]. In our context, we considered the group of residents with severe agitation as the treatment group and the group of residents with mild/no agitation as the control group. We estimated the PS for each resident using logistic regression analysis [52, 57]. Here, the severity of agitation was included as a dependent variable, and the selected matching variables were included as independent variables. After several attempts, we decided against using a distance threshold, as the random allocation within the defined threshold range resulted in more heterogeneous individuals being matched than without a defined threshold. To check the success and quality of the matching, we performed balance tests before and after the matching. In those cases where the groups were still very heterogeneous, we repeated the matching and made adjustments in the included variables or in the number of controls [52, 57]. For the balance tests, we utilised descriptive characteristics, graphs, chi-square tests and ks-tests to determine whether the balance between the two groups had improved by using the matching procedure.

After achieving a successful matching result, we determined means and standard deviations to describe the differences in the dimensions of quality of life in the matched samples. To determine the level and significance of the differences, regression models were then calculated for each of the five dimensions of quality of life. Since cluster effects caused either by the cluster of care units or by the cluster of nursing homes could not be excluded, we calculated intraclass correlation coefficients (ICC) in empty models according to Sommet et al. (2017) to select the appropriate type of regression models (linear regression models or mixed linear regression models) [58]. For the calculations of the ICC values, we used the package ‘lme4’ [59]. In the regression models, the dimensions of quality of life were the dependent variables, and the severity of agitation was the independent variable. For safety reasons (no exact matching) and to avoid further distortions, we adjusted the matching variables as control variables in the regression models [52, 57]. Depending on the results of the ICC calculations, we also included care units nested in nursing homes as a random factor in addition to the severity of agitation and the matching variables (fixed factors). Finally, to be able to provide additional information on whether the severity of the single NPI-Q items defining the construct of agitation (*agitation/aggression*, *disinhibition*, *irritability/lability*) are equally associated with the dimensions of quality of life, the selected models were calculated a second time with the three NPI-Q agitation items instead of the construct of agitation.

Since we calculated ten linear regression models, two for each dimension of quality of life, we applied Bonferroni correction from alpha=5% to alpha=0.5%. All statistical analyses were performed using R statistical software (4.0.3) [60].

## Results

The DemenzMonitor study comprises 4427 data sets from 2926 participants. Without the pretests, 4281 data sets were available for the years 2012 to 2014. According to the inclusion and exclusion criteria for the secondary data analysis, we excluded 920 data sets corresponding to participants with a DSS score ≤ 2 and 548 data sets from participants without a documented dementia diagnosis. After removing the second and third data sets of each participant (n=810), our analysis data set comprised 2003 participants, each with one measurement. To perform the statistical procedures, participants with missing values in NPI-Q items (n=2), QUALIDEM items (n=29) or variables for calculating the PS (n=25) were excluded. Accordingly, the sample prior to the matching process included 1947 participants from 66 nursing homes and 139 care units.

The prevalence of severe agitation was 6.3% (123/1947) in this sample. Of the group of residents without severe agitation, 823 residents showed mild agitation (42.3%), and 1001 residents showed no symptoms of agitation (51.4%). The participating residents with severe agitation were younger and less often female than the residents with mild or no agitation (Table 1). With regard to the length of stay in months, they had also lived for a shorter period in the nursing homes than the residents with mild or no agitation. Their DSS scores were higher, and neuropsychiatric symptoms occurred in this group more frequently overall. Regarding visits from other people, both groups received visits in over 90% of cases. The group of residents with mild or no agitation had slightly more visits than the residents with severe agitation.

**Table 1:** Characteristics of the sample subdivided by the severity of agitation

	No/Mild Agitation	Severe Agitation
Observations	1824	123
Age in years, mean (SD)	83.6 (7.8)	80.5 (10.2)
Sex, male in % (n)	22.3 (407)	31.7 (39)
DSS score (3-14), mean (SD)	9.4 (3.4)	10.5 (3.2)
PSMS score (6-30), mean (SD)	19.9 (4.9) ^*a*^	21.3 (4.3) ^*b*^
Duration of stay in months, mean (SD)	34.9 (42.6)	27.9 (38.2)
Visits, no in % (n)	3.2 (59)	6.5 (8)
Care level in % (n)		
No care dependency	0.6 (11)	1.6 (2)
1 (considerable care dependency)	23.0 (420)	18.7 (23)
2 (mild care dependency)	46.1 (840)	42.3 (52)
3 (severe care dependency)	28.7 (524)	34.2 (42)
4 (very severe care dependency)	1.6 (29)	2.4 (3)
Missing value	0 (0)	0.8 (1)
Delusions in % (n)	16.0 (292)	45.5 (56)
Anxiety in % (n)	18.4 (335)	42.3 (52)
Hallucinations in % (n)	14.0 (225)	30.1 (37)
Aberrant Motor in % (n)	30.8 (562)	62.6 (77)
Depression/Dysphoria in % (n)	26.1 (476)	43.9 (54)
Apathy/Indifference in % (n)	25.2 (459)	46.3 (57)
Euphoria/Elation in % (n)	6.7 (123)	17.1 (21)
Nighttime Disturbances in % (n)	23.7 (433) ^*c*^	49.6 (61)
Eating Disturbances in % (n)	27.5 (501) ^*d*^	46.3 (57)

Missing values: ^*a*^n=3; ^*b*^n=2; ^*c*^n=3; ^*d*^n=1; SD=standard deviation

In the PS matching, we achieved the best results with the following variables: age, sex, visit, DSS score, NPI-Q anxiety, NPI-Q hallucinations, NPI-Q delusions, NPI-Q aberrant motor and length of stay in months. The differences between the two groups regarding these variables—which were no longer significant in the final balance test—confirm that the application of the matching method was successful (Table 2). In terms of mean values and relative frequencies, we obtained two groups with very similar values in the characteristics considered relevant. The unequal prevalence of the neuropsychiatric symptoms that were not used as matching variables also decreased by matching the other variables (Table 3). The minor non-significant differences between the two groups in functionality and level of care remained after matching. Further details on the quality of the matching can be found in the additional files (Additional files 1-3).

**Table 2:** Results of the balance tests for the selected variables before and after matching

	Before Matching			After Matching		
	No/Mild Agitation	Severe Agitation	p ^*a*^	No/Mild Agitation	Severe Agitation	p ^*a*^
Observations	1824	123		246	123	
Age, mean (SD)	83.6 (7.8)	80.5 (10.2)	0.007	81.1 (8.0)	80.5 (10.2)	0.528
Sex, male % (n)	22.3 (407)	31.7 (39)	0.022	30.5 (75)	31.7 (39)	0.905
DSS score, mean (SD)	9.4 (3.4)	10.5 (3.2)	<0.001	10.5 (3.0)	10.5 (3.2)	0.921
Duration of stay, mean (SD)	34.9 (42.6)	27.9 (38.2)	0.060	27.4 (25.7)	27.9 (38.2)	0.416
Visits, no % (n)	3.2 (59)	6.5 (8)	0.095	6.1 (15)	6.5 (8)	1.000
Delusions, % (n)	16.0 (292)	45.5 (56)	<0.001	45.5 (112)	45.5 (56)	1.000
Anxiety, % (n)	18.4 (335)	42.3 (52)	<0.001	42.3 (104)	42.3 (52)	1.000
Hallucinations, % (n)	14.0 (225)	30.1 (37)	<0.001	24.0 (59)	30.1 (37)	0.257
Aberrant Motor, % (n)	30.8 (562)	62.6 (77)	<0.001	66.3 (163)	62.6 (77)	0.562

^*a*^Calculated using chi-square tests or ks-tests; SD=standard deviation

**Table 3:** Differences in the prevalence of the variables that were not matched before and after matching

	Before Matching			After Matching		
	No/Mild Agitation	Severe Agitation	p ^*a*^	No/Mild Agitation	Severe Agitation	p ^*a*^
Observations	1824	123		246	123	
PSMS score, mean (SD)	19.9 (4.9)	21.3 (4.3)	0.003	20.0 (4.5)	21.3 (4.3)	0.040
Care level in % (n)						
No care dependency	0.6 (11)	1.6 (2)	0.308	0 (0)	1.6 (2)	0.095
1 (considerable care dependency)	23.0 (420)	18.7 (23)		20.3 (50)	18.7 (23)	
2 (mild care dependency)	46.1 (840)	42.3 (52)		47.6 (117)	42.3 (52)	
3 (severe care dependency)	28.7 (524)	34.2 (42)		31.7 (78)	34.2 (42)	
4 (very severe care dependency)	1.6 (29)	2.4 (3)		0.4 (1)	2.4 (3)	
Missing value	0 (0)	0.8 (1)		0 (0)	0.8 (1)	
Depression/Dysphoria, % (n)	26.1 (476)	43.9 (54)	<0.001	40.2 (99)	43.9 (54)	0.575
Apathy/Indifference, % (n)	25.2 (459)	46.3 (57)	<0.001	36.6 (90)	46.3 (57)	0.091
Euphoria/Elation, % (n)	6.7 (123)	17.1 (21)	<0.001	13.0 (32)	17.1 (21)	0.372
Nighttime Disturbances, % (n)	23.7 (433) ^*b*^	49.6 (61)	<0.001	40.7 (100)	49.6 (61)	0.128
Eating Disturbances, % (n)	27.5 (501) ^*c*^	46.3 (57)	<0.001	31.3 (77)	46.3 (57)	0.007

^*a*^Calculated using chi-square tests or ks-tests; Missing values: ^*b*^n=3; ^*c*^n=1; SD=standard deviation

**Fig. 1:**
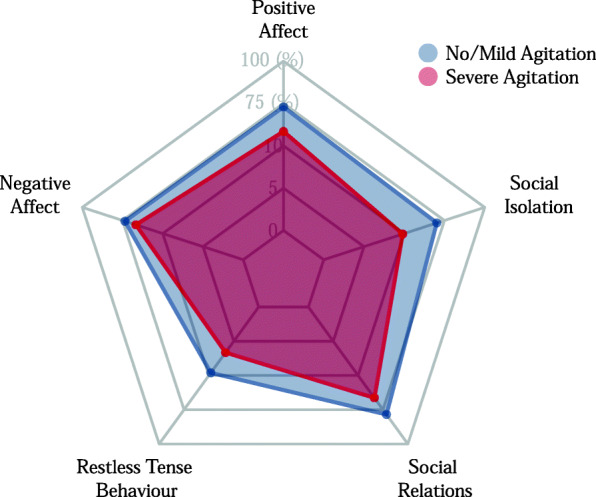
Results in dimensions of quality of life. Comparison of the mean values for residents with severe agitation and residents with no or mild agitation of the single dimensions of quality of life of the short version of QUALIDEM

After matching, the sub-sample comprised 369 participants from 64 nursing homes and 115 care units. In the descriptive evaluation, we found differences in the mean values of the single dimensions of quality of life between both groups of the matched sample. Compared with the group of residents with mild or no agitation, residents with severe agitation had lower mean values in all dimensions of quality of life (Fig. 1).

The ICC calculations in the empty models of the single dimensions of quality of life led to the following results. In the dimensions *positive affect* (0.178), *negative affect* (0.184), and *restless tense behaviour* (0.178), approximately 20% of the variance could be explained by the clusters (care units nested in nursing homes), while in the dimensions *social relations* (0.077) and *social isolation* (0.126) approximately 10% could be explained by the clusters. We therefore decided to compute mixed linear models to address the cluster effect. In the mixed linear regression models, we found significant differences between both agitation groups in the dimensions *positive affect*, *restless tense behaviour*, *social relations* and *social isolation* (p <0.001) (Table ??). To compare the coefficients of the severity of agitation of the different mixed linear regression models, we calculated the percentages of the coefficients in relation to the scale size of the respective dimension of quality of life. Converted to the scale size, we found the greatest difference between residents with severe agitation and the group of residents with mild/no agitation in the dimension *social isolation* with 23.0% (−2.07 (95% CI: −2.57,−1.57)). The negative coefficient shows that the values of quality of life decrease with an increase in agitation, that is, from no/mild agitation to severe agitation. The dimension *restless tense behaviour* had the second largest difference with a coefficient of −1.52 (95% CI: −2.04,−1.00)). This corresponds to a percentage difference of 16.9%. The dimension *positive affect* (−1.68 (95% CI: −2.28,−1.09)) showed similar results with a difference of approximately 14.0%, while the difference was smallest in the dimension *social relations* (−1.12 (95% CI: −1.54,−0.71)) with 12.4%. We did not find any difference between the two groups in the dimension *negative affect* (−0.30 (95% CI: −0.63, 0.03)) (p >0.01). The comparison of all coefficients in the single regression models illustrates that in the dimensions *social isolation*, *positive affect* and *social relations*, only the coefficient for the severity of agitation was significant. In the dimension *restless tense behaviour*, we found a significant relationship with the DSS score and the variables NPI-Q aberrant motor and NPI-Q anxiety. Here, the relation with the variable NPI-Q aberrant motor was greater than the relation with the severity of agitation. The complete mixed linear regression models with all coefficients can be found in the additional files (Additional file 4).

In the mixed linear regression models with the single NPI-Q items defining the construct of agitation, we found a significant relationship between the severity of the item *agitation/aggression* and the dimensions *positive affect, restless tense behaviour, social relations* and *social isolation*. The severity of the item *irritability* was significantly associated with the dimensions *positive affect, social relations* and *social isolation*, while the severity of the item *disinhibition* was only significantly associated with the dimension *social isolation* (Table 4, Additional file 5).

**Table 4:** Coefficients of the single NPI-Q agitation items of the adjusted ^*a*^ mixed linear regression models

Dependent variable	Agitation/Aggression ^*b*^	Disinhibition ^*b*^	Irritability/Lability ^*b*^
Quality of life dimension *positive affect*	-1.53 ***	-0.28	-1.36 **
Quality of life dimension *negative affect*	-0.20	-0.08	-0.08
Quality of life dimension *restless tense behaviour*	-1.21 ***	-0.47	-0.74
Quality of life dimension *social relations*	-1.02 ***	0.30	-1.12 ***
Quality of life dimension *social isolation*	-1.47 ***	-1.52 ***	-1.10 **

^*a*^Adjusted for age, sex, visit, length of stay in months, delusions, anxiety, aberrant motor, hallucinations;

^*b*^Coefficient for severity of the NPI-Q item (changes in quality of life when moving from no/mild to severe)

Random factor = care units nested in nursing homes; SE=standard error; CI=confidence interval

^***^p <0.001, ** *p* <0.005

## Discussion

We found that residents with dementia with severe agitation differ from residents with dementia with mild or no agitation; they tend to be younger, more often male, have stronger cognitive impairments and more neuropsychiatric symptoms than the comparison group. In the matched sample, the findings confirm that residents with dementia with severe agitation also have a significantly lower quality of life in the dimensions *positive affect*, *restless tense behaviour*, *social relations* and *social isolation* than the group of residents with dementia with mild or no agitation. Considering the differences in the individual items that define the construct of agitation, we also found that the severity of the item *agitation/aggression* is associated with the same dimensions of quality of life as the severity of the construct of agitation, while the severity of the item *irritability* is not associated with the dimension *restless tense behaviour* and the severity of the item *disinhibition* is only associated with the dimension *social isolation*. It can therefore be assumed that severe agitation is associated with lower values in four out of five dimensions of quality of life of the short version of the QUALIDEM instrument and that the individual NPI-Q items that define the construct of agitation influence these correlations to different degrees.

When investigating the characteristics of residents with very frequent agitation, Veldwijk-Rouwenhorst et al. (2017) also found differences in age, cognitive impairment and neuropsychiatric symptoms among residents with varying degrees of agitation [8]. In terms of sex distribution, their study did not find a difference between the two groups observed. The selection of nursing home units in their study and the DemenzMonitor study may be one reason for the different results. While the DemenzMonitor study included all care units that agreed to participate [39], the data set used by Veldwijk-Rouwenhorst et al. (2017) was based solely on dementia-specific care units [8]. In addition, the different definitions and measurements of severe or very frequent agitation may have influenced the allocation to the different groups.

Since we did not find any study that examined the difference between residents with severe agitation and residents with mild or no agitation in the different dimensions of quality of life, our results can only be compared with those of studies that did not make any classification regarding the severity of agitation. The significant relationships between agitation and the dimensions *positive affect* [27, 29, 34], *restless tense behaviour* [27, 29, 34] and *social isolation* [27, 29], and the absence of a relationship between agitation and the dimension *negative affect* [27, 34] are supported by other studies that did not focus on residents with severe agitation but on agitation in general. However, in contrast to our results and those of van Kooten et al. (2017), neither Gräske et al. (2014) nor Henskens et al. (2019) found a significant relationship between the dimension *social relations* and agitation. Divergent concepts of agitation, smaller samples in the studies of Gräske et al. (2014) and Henskens et al. (2019), and different inclusion and exclusion criteria—which led to the samples of Gräske et al. (2014) and Henskens et al. (2019) showing better values of cognition and functionality—could explain the different results in this dimension [27, 29, 34]. As these three studies defined agitation with the CMAI [27, 29] or a composite score of the NPI items *agitation/aggression, disinhibition, irritability/lability* and *euphoria/elation* [34], the results for the individual items of the construct of agitation cannot be compared with other studies.

The difference in the dimension *social isolation* could be explained by the item *calls* and the strategies that carers use to react to agitation. Although vocalisations are not included in the agitation items used here, they are, according to common definitions, a symptom of agitation that increases with severe agitation [11, 12]. At the same time, severe agitation represents a challenge for caregivers [61]. Rapaport et al. (2018) found that caregivers perceive loud and repeated shouting as intentionally demanding and that they associate it with a desire for attention [62]. A German study by Höwler (2011) confirms that showing severe agitation is perceived as a crisis by nursing staff. Interventions used by nurses aim to de-escalate the situation, resulting in spatial and social isolation [63]. In a quantitative study by Cooper et al. (2018), in which 1544 employees of nursing homes in England were interviewed, approximately 25% of the respondents also stated that they sometimes consciously avoid a person with agitated behaviour to prevent stress [64]. Assuming that the other residents of the care facility are disturbed or stressed by the behaviour [62, 63] and that severe disinhibition is also characterised by offensive language used by the resident, this may additionally explain why residents with severe agitation were more often rejected by other residents.

The difference in the dimension *restless tense behaviour* could be explained by the items of restlessness (*is restless; makes restless movements*). In the definition of agitation by Cohen-Mansfield et al. (1989), frequent wandering is described as a major symptom of agitation [11]. Regier and Gitlin (2018) confirm that pronounced dementia-related restlessness, as measured by the Agitated Behaviours in Dementia Scale, can be associated with higher pain levels, the administration of medication and the demonstration of severe agitation. They conclude that although restlessness should not be subsumed under the term agitation, it is a distinct form of agitation that has a negative impact on the well-being of people with dementia and their carers [65]. The assumption that restlessness could explain the relation between the severity of agitation and this dimension is also supported by the fact that the NPI-Q item aberrant motor in this regression model demonstrates a stronger relation with this quality-of-life dimension (Additional file 4) and that the severity of the items *irritability* and *disinhibition* is furthermore not associated with this dimension. A cluster-randomised controlled study by Husebo et al. (2014) showed that systematic pain management can reduce agitation and the associated restlessness [66]. Since the presence of pain was not recorded in the DemenzMonitor study [39], a possible bias in the results due to an increased pain sensation in the group of residents with severe agitation cannot be excluded.

The difference in the dimension *positive affect* could be explained by the fact that agitation in general is hard to treat. Especially in cases of severe agitation, difficulties in treatment lead to people with severe agitation being restrained or treated with psychotropic drugs [67, 68]. However, even these interventions cannot reduce agitated behaviour beyond a certain level [6, 67]. Furthermore, an analysis by Henskens et al. (2019) showed that the variance in the values of the dimension *positive affect* can most likely be explained by the neuropsychiatric symptoms apathy and depression [29]. By performing PS matching, we were able to reduce the differences in the prevalence of apathy between the two groups, but residents with severe agitation still express apathy more often overall (Table 3). Both of these reasons could explain why residents with severe agitation have a lower quality of life in this dimension than residents with mild or no agitation.

Livingston et al. (2014) showed in their review that although a relationship between agitation and quality of life is assumed, interventions that reduced agitation still had no effect on the overall score of quality of life. They therefore recommended focusing on the single dimensions of quality of life [37]. According to our results, an intervention aimed at both reducing the severity of agitation and improving quality of life should focus on the dimensions *social isolation, restless tense behaviour, positive affect,* and *social relations*. To avoid social isolation, it is necessary to understand the causes of agitated behaviour and to learn alternative strategies for addressing acute and challenging situations [62]. In this context, training of person-centred care, promotion of social interactions and person-centred activities, and education in the use of antipsychotic medications could be useful for nurses. Increased participation in activities could furthermore be associated with better values in the dimensions *restless tense behaviour, social relations* and *positive affect* of quality of life [69]. Knowledge acquisition and further training could additionally be helpful for identifying triggers of acute behaviour at an early stage, for reducing spatial and social isolating interventions and for replacing them with person-centred approaches [70]. However, to empower nurses, it will be necessary not only to record the nurses’ competencies in terms of attitudes, knowledge and skills but also to observe how knowledge, skills and strategies are used and applied in daily practice.

### Limitations

The DemenzMonitor study itself has some limitations. Since it was based on a convenience sample, the representativeness of the results is limited. The matching procedure also reduced the generalisability of the results: after matching, the characteristics of the analysed sub-sample no longer matched those of residents with dementia in German nursing homes in general. By using secondary data, only the variables collected in the primary study could be used for matching. Distortions of the results caused by other factors that were not assessed in the DemenzMonitor study, such as the presence of pain or the intake of medication, could not be eliminated. In addition, only one measurement point per person was included in the analysis data set. Statements on the causal relationships between the severity of agitation and the single dimensions of quality of life are therefore not possible.

Another important point is the method of data collection (proxy assessment by different caregivers). The proxy assessment of quality of life is recommended for people with severe dementia [19, 71]. Nevertheless, proxy assessment of quality of life could be associated with caregiver burden [72]. Since severe agitation is considered very challenging and stressful, both the proxies’ assessment of agitation and quality of life could have been biased by caregiver burden. On the other hand, Hongisto et al. (2018) concluded in their study that a self-assessed quality of life of people with dementia should not be used to test neuropsychiatric symptoms because the symptoms are not perceived by individuals with dementia themselves [73]. This controversy has not been resolved to date and should be addressed in future research.

The definition of the construct of agitation may also have distorted the results. Since the NPI-Q was not primarily developed to assess agitation, the definition of the construct of agitation enabled the combination of individual aspects of agitation (aggression, cooperation and rejection, impulsive behaviour, verbal abuse and impatience or irritability) into one score, resulting in a measurable conceptualisation of agitation that did not reduce agitation only to uncooperative and rejective behaviour (item *agitation/aggression*). Although Palm et al. (2018), Reuther et al. (2016) and Selbæk and Engedal (2012) confirm that the items *agitation/aggression*, *disinhibition* and *irritability/lability* are correlated [9, 49, 50], we found that the items are not equally associated with the dimensions of quality of life and could therefore indicate other non-pharmacological or pharmacological therapies. Finally, when interpreting the results, researchers should keep in mind the explorative character of the analysis, the small number of items included in the QUALIDEM instrument and the overlaps with the definitions of agitation.

## Conclusions

The results of the secondary data analysis show that residents with dementia with severe agitation and residents with dementia with mild or no agitation differ not only in demographic characteristics and the presence of neuropsychiatric symptoms but also in certain dimensions of quality of life. Residents with dementia with severe agitation exhibit a significantly lower quality of life in four of the five dimensions of the QUALIDEM instrument. Interventions that aim to influence both the severity of agitation and the quality of life as a measure of outcome should focus on the specific dimensions of quality of life that are correlated with agitation. In future intervention studies, it would also be helpful to 1) specify whether the residents exhibit only individual behavioural symptoms such as disinhibition or a combination of agitation symptoms to address the relevant dimensions of quality of life and 2) to no longer use the total score of all dimensions of quality of life but a newly formed score corresponding to those dimensions that differ among people with severe agitation as a measure of outcome and to examine this score with regard to the effectiveness of the interventions. The assessments of single dimensions of quality of life could thus be more easily understood by nurses and help them to directly reflect applied interventions and their consequences in their daily work.

## Supplementary Information

**Additional file 1** Distribution of propensity scores. Representation of the distribution of propensity scores divided into 1) groups of participants with severe agitation and with mild or no agitation and 2) groups of matched and unmatched participants.

**Additional file 2** Density of propensity scores before matching. Comparison of participants with severe agitation with those with mild or no agitation before matching.

**Additional file 3** Density of propensity scores after matching. Comparison of participants with severe agitation with those with mild or no agitation after matching.

**Additional file 4** Mixed linear regression models with all coefficients. Mixed linear regression models for the dimensions positive affect, negative affect, restless tense behaviour, social relations and social isolation as dependent variables; the severity of agitation and the matching variables as fixed factors; and the care units nested in nursing homes as random factor with all measured coefficients.

**Additional file 5** Mixed linear regression models with the single NPI-Q items defining the construct of agitation. Mixed linear regression models for the dimensions positive affect, negative affect, restless tense behaviour, social relations and social isolation as dependent variables; the severity of each NPI-Q item defining the construct of agitation and the matching variables as fixed factors; and the care units nested in nursing homes as random factor with all measured coefficients.

## Data Availability

The data sets generated and/or analysed during the current study are not publicly available due to the fact that they involve person-related data subject to the legal regulations of the German Center for Neurodegenerative Diseases, but are available from the corresponding author on reasonable request. Declarations

## Abbreviations

CMAI: Cohen-Mansfield agitation inventory
DSS: Dementia screening scale
IPA: International psychogeriatric association
ICC: Intraclass correlation coefficient
NPI: Neuropsychiatric inventory
NPI-Q: Neuropsychiatric inventory questionnaire
PS: Propensity score
PSMS: Physical self-maintenance scale
