# Supplementary figures and images for: Relationship between the severity of agitation and quality of life in residents with dementia living in German nursing homes - a secondary data analysis
Source: BMC Psychiatry. 2021 Apr 13;21:191. doi: 10.1186/s12888-021-03167-5 (PMC8042694; doi:10.1186/s12888-021-03167-5)

# Distribution of Propensity Scores

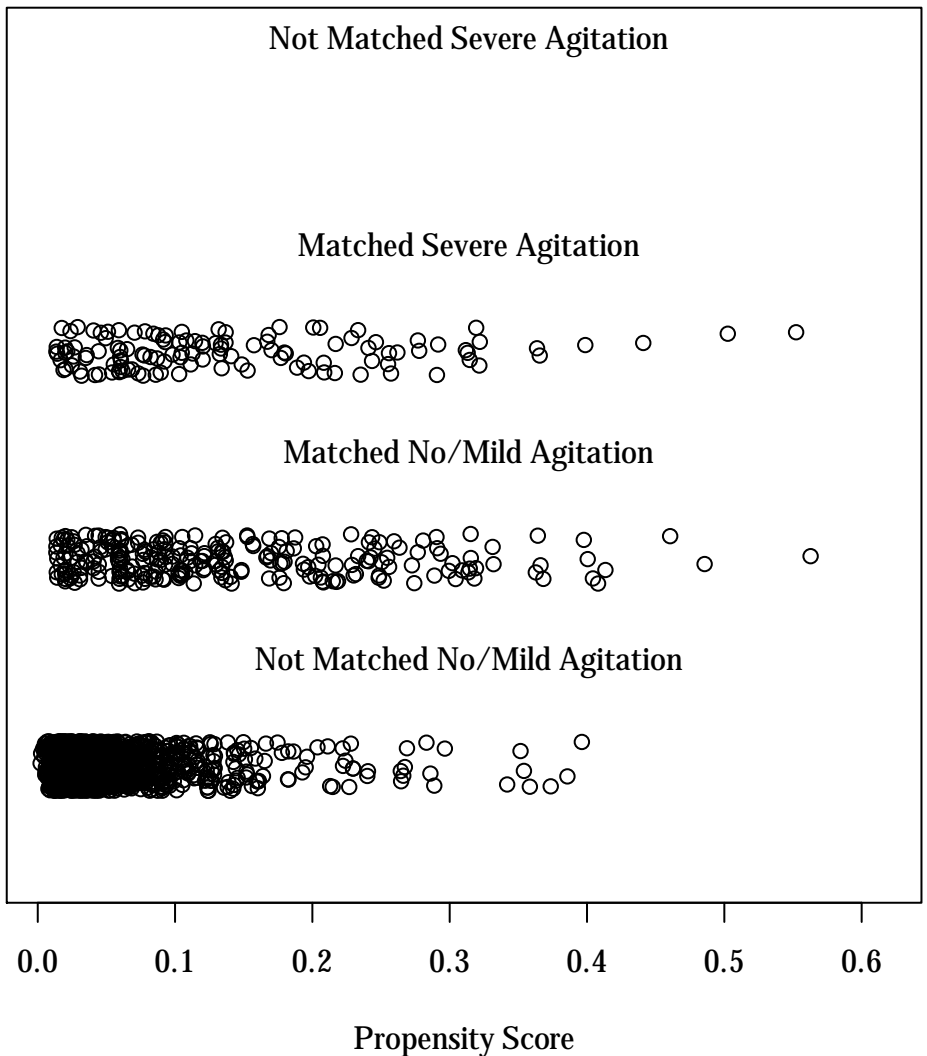

Supplement: Supplementary file 1 — Additional file 1 Distribution of propensity scores. Representation of the distribution of propensity scores divided into 1) groups of participants with severe agitation and with mild or no agitation and 2) groups of matched and unmatched participants. [file 12888_2021_3167_MOESM1_ESM.pdf]

# Density of Propensity Scores before matching

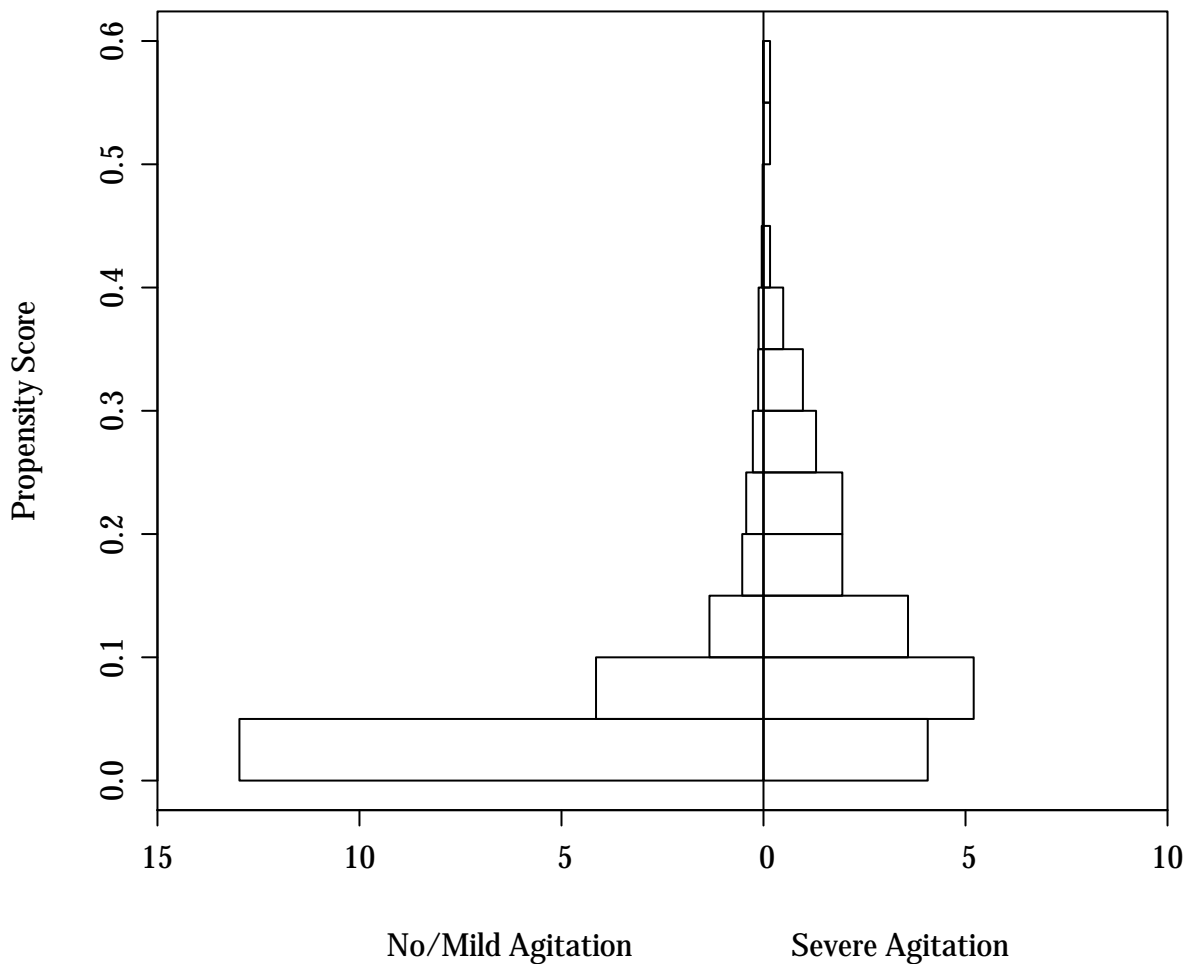

Supplement: Supplementary file 2 — Additional file 2 Density of propensity scores before matching. Comparison of participants with severe agitation with those with mild or no agitation before matching. [file 12888_2021_3167_MOESM2_ESM.pdf]

# Density of Propensity Scores after matching

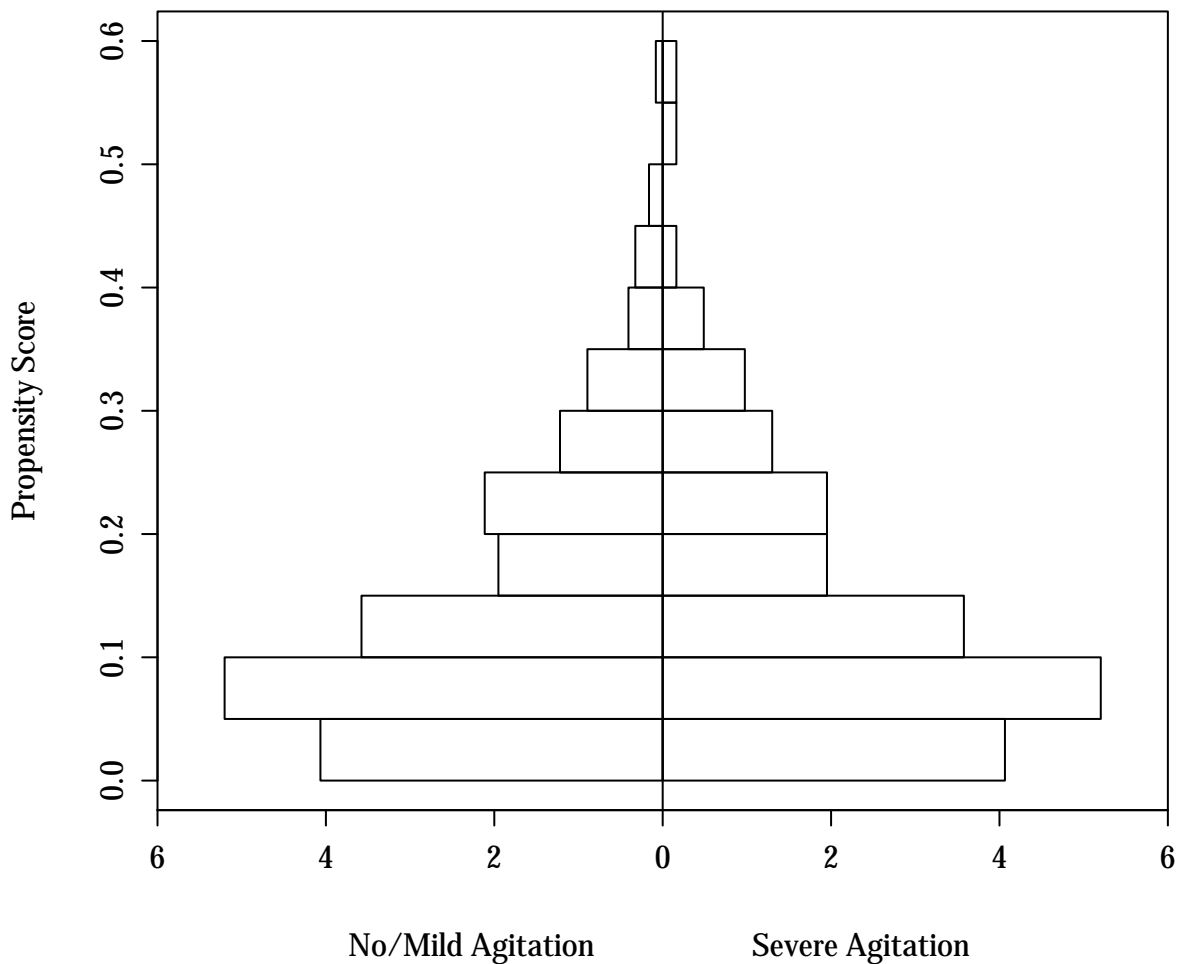

Supplement: Supplementary file 3 — Additional file 3 Density of propensity scores after matching. Comparison of participants with severe agitation with those with mild or no agitation after matching. [file 12888_2021_3167_MOESM3_ESM.pdf]
