# Supplementary material for: Relationship between the severity of agitation and quality of life in residents with dementia living in German nursing homes - a secondary data analysis
Source: BMC Psychiatry. 2021 Apr 13;21:191. doi: 10.1186/s12888-021-03167-5 (PMC8042694; doi:10.1186/s12888-021-03167-5)
Supplement: Supplementary file 4 — Additional file 4 Mixed linear regression models with all coefficients. Mixed linear regression models for the dimensions positive affect, negative affect, restless tense behaviour, social relations and social isolation as dependent variables; the severity of agitation and the matching variables as fixed factors; and the care units nested in nursing homes as random factor with all measured coefficients. [file 12888_2021_3167_MOESM4_ESM.pdf]

Additional file 4: Mixed linear regression models with all coefficients

Table S1 Mixed linear regression model for dimension *positive affect* as dependent variable, the severity of agitation and the matching variables as fixed factors and care units nested in nursing homes as random factor

Independent Variables (fixed effects)	Coefficients	95% CI	SE	t	p
**(Intercept)**	**9.81**	**7.06, 12.59**	**1.42**	**6.90**	**<0.001**
**Severe Agitation (yes)**	**-1.68**	**-2.28, -1.09**	**0.31**	**-5.51**	**<0.001**
Age	0.01	-0.03, 0.04	0.02	0.29	0.775
Sex (female)	-0.32	-0.94, 0.29	0.31	-1.04	0.301
Visits (no)	-0.53	-1.68, 0.62	0.59	-0.90	0.371
DSS score	-0.14	-0.24, -0.04	0.05	-2.80	0.005
Duration of stay	0.01	<-0.01, 0.01	0.01	1.20	0.232
NPI-Q Delusions (no)	0.12	-0.46, 0.71	0.30	0.41	0.683
NPI-Q Anxiety (no)	-0.21	-0.80, 0.38	0.30	-0.69	0.492
NPI-Q Aberrant Motor (no)	-0.66	-1.31, -0.03	0.33	-2.03	0.043
NPI-Q Hallucinations (no)	0.43	-0.24, 1.10	0.34	1.26	0.208

Random effects: Care unit (intercept): Variance = 0.59; SD = 0.77; .sig01 = 0.00, 1.46;
Nursing home (intercept): Variance = 0.56; SD = 0.75; .sig02 = 0.00, 1.29;
Residual: Variance = 6.18; SD = 2.49; .sigma = 2.25, 2.68;
Number of obs.: 369, Care units: 115; Nursing homes: 64

Table S2 Mixed linear regression model for dimension *negative affect* as dependent variable, the severity of agitation and the matching variables as fixed factors and care units nested in nursing homes as random factor

Independent Variables (fixed effects)	Coefficients	95% CI	SE	t	p
**(Intercept)**	**2.78**	**1.27, 4.29**	**0.78**	**3.58**	**<0.001**
Severe Agitation (yes)	-0.30	-0.63, 0.03	0.17	-1.78	0.075
Age	0.02	<0.01, 0.04	0.01	2.01	0.045
**Sex (female)**	**-0.86**	**-1.19, -0.53**	**0.17**	**-5.08**	**<0.001**
Visits (no)	0.44	-0.20, 1.07	0.33	1.34	0.182
DSS score	-0.03	-0.09, 0.02	0.03	-1.21	0.227
Duration of stay	<-0.01	-0.01, <0.01	<0.01	-0.60	0.549
NPI-Q Delusions (no)	0.12	-0.20, 0.44	0.17	0.71	0.478
**NPI-Q Anxiety (no)**	**1.23**	**0.91, 1.55**	**0.16**	**7.49**	**<0.001**
NPI-Q Aberrant Motor (no)	-0.10	-0.45, 0.24	0.18	-0.58	0.562
NPI-Q Hallucinations (no)	0.35	-0.01, 0.72	0.19	1.88	0.062

Random effects: Care unit (intercept): Variance = 0.08; SD = 0.27; .sig01 = 0.00, 0.68;
Nursing home (intercept): Variance = 0.43; SD = 0.66; .sig02 = 0.27, 0.91;
Residual: Variance = 1.80; SD = 1.34; .sigma = 1.22, 1.44;
Number of obs.: 369, Care units: 115; Nursing homes: 64

Table S3 Mixed linear regression model for dimension *restless tense behaviour* as dependent variable, the severity of agitation and the matching variables as fixed factors and care units nested in nursing homes as random factor

Independent Variables (fixed effects)	Coefficients	95% CI	SE	t	p
(Intercept)	2.33	-0.04, 4.72	1.22	1.91	0.057
**Severe Agitation (yes)**	**-1.52**	**-2.04, -1.00**	**0.27**	**-5.72**	**<0.001**
Age	0.02	-0.01, 0.05	0.01	1.31	0.191
Sex (female)	0.58	0.06, 1.11	0.27	2.18	0.030
Visits (no)	0.22	-0.76, 1.20	0.50	0.43	0.670
**DSS score**	**-0.13**	**-0.22, -0.05**	**0.04**	**-3.10**	**0.002**
Duration of stay	<0.01	<-0.01, 0.01	<0.01	0.97	0.331
NPI-Q Delusions (no)	0.10	-0.40, 0.60	0.26	0.40	0.692

Independent Variables (fixed effects)	Coefficients	95% CI	SE	t	p
**NPI-Q Anxiety (no)**	**1.11**	**0.60, 1.62**	**0.26**	**4.26**	**<0.001**
**NPI-Q Aberrant Motor (no)**	**1.73**	**1.19, 2.28**	**0.28**	**6.17**	**<0.001**
NPI-Q Hallucinations (no)	0.11	-0.46, 0.69	0.30	0.38	0.704

Random effects: Care unit (intercept): Variance = 1.15; SD = 1.07; .sig01 = 0.58, 1.52;
Nursing home (intercept): Variance = 0.29; SD = 0.54; .sig02 = 0.00, 1.12;
Residual: Variance = 4.31; SD = 2.08; .sigma = 1.88, 2.23;
Number of obs.: 369, Care units: 115; Nursing homes: 64

Table S4 Mixed linear regression model for dimension *social relations* as dependent variable, the severity of agitation and the matching variables as fixed factors and care units nested in nursing homes as random factor

Independent Variables (fixed effects)	Coefficients	95% CI	SE	t	p
**(Intercept)**	**6.57**	**4.66, 8.50**	**0.99**	**6.65**	**<0.001**
**Severe Agitation (yes)**	**-1.12**	**-1.54, -0.71**	**0.21**	**-5.28**	**<0.001**
Age	<0.01	-0.02, 0.03	0.01	0.27	0.790
Sex (female)	0.28	-0.14, 0.70	0.22	1.29	0.197
Visits (no)	-0.49	-1.29, 0.31	0.41	-1.18	0.239
DSS score	0.01	-0.06, 0.07	0.03	0.14	0.890
Duration of stay	0.01	<-0.01, 0.01	<0.01	1.64	0.101
NPI-Q Delusions (no)	-0.06	-0.47, 0.35	0.21	-0.27	0.784
NPI-Q Anxiety (no)	-0.56	-0.97, -0.15	0.21	-2.68	0.008
NPI-Q Aberrant Motor (no)	-0.53	-0.97, -0.09	0.23	-2.34	0.020
NPI-Q Hallucinations (no)	0.59	0.13, 1.05	0.24	2.47	0.014

Random effects: Care unit (intercept): Variance = 0.25; SD = 0.50; .sig01 = 0.00, 0.99;
Nursing home (intercept): Variance = 0.23; SD = 0.48; .sig02 = 0.00, 0.94;
Residual: Variance = 3.02; SD = 1.74; .sigma = 1.57, 1.87;
Number of obs.: 369, Care units: 115; Nursing homes: 64

Table S5 Mixed linear regression model for dimension *social isolation* as dependent variable, the severity of agitation and the matching variables as fixed factors and care units nested in nursing homes as random factor

Independent Variables (fixed effects)	Coefficients	95% CI	SE	t	p
**(Intercept)**	**7.65**	**5.37, 9.94**	**1.18**	**6.50**	**<0.001**
**Severe Agitation (yes)**	**-2.07**	**-2.57, -1.57**	**0.25**	**-8.14**	**<0.001**
Age	-0.02	-0.05, <0.01	0.01	-1.72	0.086
Sex (female)	0.13	-0.37, 0.63	0.26	0.50	0.618
Visits (no)	0.27	-0.67, 1.22	0.49	0.56	0.575
DSS score	-0.05	-0.13, 0.03	0.04	-1.18	0.238
Duration of stay	<0.01	-0.01. 0.01	<0.01	0.24	0.813
NPI-Q Delusions (no)	0.45	-0.04, 0.94	0.25	1.80	0.072
NPI-Q Anxiety (no)	0.69	0.21, 1.18	0.25	2.77	0.006
NPI-Q Aberrant Motor (no)	0.37	-0.15, 0.90	0.27	1.38	0.168
NPI-Q Hallucinations (no)	0.38	-0.17, 0.93	0.29	1.34	0.180

Random effects: Care unit (intercept): Variance = 0.48; SD = 0.70; .sig01 = 0.00, 1.24;
Nursing home (intercept): Variance = 0.50; SD = 0.71; .sig02 = 0.00, 1.15;
Residual: Variance = 4.14; SD = 2.04; .sigma = 1.85, 2.19;
Number of obs.: 369, Care units: 115; Nursing homes: 64
