# Supplementary material for: Relationship between the severity of agitation and quality of life in residents with dementia living in German nursing homes - a secondary data analysis
Source: BMC Psychiatry. 2021 Apr 13;21:191. doi: 10.1186/s12888-021-03167-5 (PMC8042694; doi:10.1186/s12888-021-03167-5)
Supplement: Supplementary file 5 — Additional file 5 Mixed linear regression models with the single NPI-Q items defining the construct of agitation. Mixed linear regression models for the dimensions positive affect, negative affect, restless tense behaviour, social relations and social isolation as dependent variables; the severity of each NPI-Q item defining the construct of agitation and the matching variables as fixed factors; and the care units nested in nursing homes as random factor with all measured coefficients. [file 12888_2021_3167_MOESM5_ESM.pdf]

Additional file 5: Mixed linear regression models with the single NPI-Q items defining the construct of agitation

Table S1 Mixed linear regression model for dimension *positive affect* as dependent variable, the severity of each NPI-Q item defining the construct of agitation and the matching variables as fixed factors and care units nested in nursing homes as random factor

Independent Variables (fixed effects)	Coefficients	95% CI	SE	t	p
**(Intercept)**	**9.64**	**6.91, 12.40**	**1.42**	**6.80**	**<0.001**
**NPI-Q Severe Agitation/Aggression (yes)**	**-1.53**	**-2.24, -0.83**	**0.36**	**-4.20**	**<0.001**
NPI-Q Severe Disinhibition (yes)	-0.28	-1.23, 0.68	0.49	-0.56	0.575
**NPI-Q Severe Irritability/Lability (yes)**	**-1.36**	**-2.16, -0.55**	**0.42**	**-3.24**	**0.001**
Age	0.01	-0.03, 0.04	0.02	0.45	0.654
Sex (female)	-0.36	-0.97, 0.25	0.31	-1.16	0.246
Visits (no)	-0.71	-1.87, 0.44	0.60	-1.19	0.234
DSS score	-0.14	-0.24, -0.04	0.05	-2.61	0.009
Duration of stay	0.01	-0.01, 0.01	0.01	0.97	0.333
NPI-Q Delusions (no)	-0.02	-0.60, 0.57	0.30	-0.05	0.960
NPI-Q Anxiety (no)	-0.24	-0.82, 0.34	0.30	-0.79	0.430
NPI-Q Aberrant Motor (no)	-0.70	-1.34, -0.07	0.33	-2.16	0.031
NPI-Q Hallucinations (no)	0.49	-0.18, 1.51	0.34	1.43	0.153

Random effects: Care unit (intercept): Variance = 0.41; SD = 0.64; .sig01 = 0.00, 1.38;
Nursing home (intercept): Variance = 0.63; SD = 0.80; .sig02 = 0.00, 1.31;
Residual: Variance = 6.17; SD = 2.48; .sigma = 2.24, 2.67;
Number of obs.: 369, Care units: 115; Nursing homes: 64

Table S2 Mixed linear regression model for dimension *negative affect* as dependent variable, the severity of each NPI-Q item defining the construct of agitation and the matching variables as fixed factors and care units nested in nursing homes as random factor

Independent Variables (fixed effects)	Coefficients	95% CI	SE	t	p
**(Intercept)**	**2.73**	**1.22, 4.25**	**0.78**	**3.50**	**<0.001**
NPI-Q Severe Agitation/Aggression (yes)	-0.20	-0.59, 0.19	0.20	-1.00	0.317
NPI-Q Severe Disinhibition (yes)	-0.08	-0.61, 0.45	0.27	-0.30	0.761
NPI-Q Severe Irritability/Lability (yes)	-0.08	-0.53, 0.37	0.23	-0.35	0.730
Age	0.02	<0.01, 0.04	0.01	1.97	0.050
**Sex (female)**	**-0.86**	**-1.19, -0.53**	**0.17**	**-5.07**	**<0.001**
Visits (no)	0.43	-0.21, 1.07	0.33	1.30	0.196
DSS score	-0.03	-0.09, 0.02	0.03	-1.08	0.281
Duration of stay	<-0.01	-0.01, <0.01	<0.01	-0.64	0.522
NPI-Q Delusions (no)	0.10	-0.23, 0.42	0.17	0.59	0.556
**NPI-Q Anxiety (no)**	**1.23**	**0.91, 1.55**	**0.17**	**7.42**	**<0.001**
NPI-Q Aberrant Motor (no)	-0.11	-0.46, 0.24	0.18	-0.61	0.546
NPI-Q Hallucinations (no)	0.37	<0.01, 0.73	0.19	1.95	0.052

Random effects: Care unit (intercept): Variance = 0.06; SD = 0.25; .sig01 = 0.00, 0.67;
Nursing home (intercept): Variance = 0.45; SD = 0.67; .sig02 = 0.29, 0.93;
Residual: Variance = 1.82; SD = 1.35; .sigma = 1.22, 1.44;
Number of obs.: 369, Care units: 115; Nursing homes: 64

Table S3 Mixed linear regression model for dimension *restless tense behaviour* as dependent variable, the severity of each NPI-Q item defining the construct of agitation and the matching variables as fixed factors and care units nested in nursing homes as random factor

Independent Variables (fixed effects)	Coefficients	95% CI	SE	t	p
(Intercept)	2.15	-0.25, 4.56	1.24	1.73	0.084
**NPI-Q Severe Agitation/Aggression (yes)**	**-1.21**	**-1.83, -0.59**	**0.32**	**-3.79**	**<0.001**
NPI-Q Severe Disinhibition (yes)	-0.47	-1.30, 0.37	0.43	-1.08	0.282
NPI-Q Severe Irritability/Lability (yes)	-0.74	-1.46, -0.04	0.36	-2.04	0.042
Age	0.02	-0.01, 0.05	0.01	1.33	0.184

Independent Variables (fixed effects)	Coefficients	95% CI	SE	t	p
Sex (female)	0.56	0.04, 1.09	0.27	2.07	0.040
Visits (no)	0.14	-0.86, 1.15	0.52	0.28	0.781
DSS score	-0.12	-0.21, -0.04	0.05	-2.75	0.006
Duration of stay	<0.01	-0.01, 0.01	<0.01	0.74	0.460
NPI-Q Delusions (no)	-0.01	-0.52, 0.50	0.26	-0.03	0.973
**NPI-Q Anxiety (no)**	**1.09**	**0.58, 1.61**	**0.26**	**4.13**	**<0.001**
**NPI-Q Aberrant Motor (no)**	**1.70**	**1.14, 2.25**	**0.29**	**5.94**	**<0.001**
NPI-Q Hallucinations (no)	0.17	-0.40, 0.75	0.30	0.58	0.561

Random effects: Care unit (intercept): Variance = 0.96; SD = 0.98; .sig01 = 0.45, 1.46;
Nursing home (intercept): Variance = 0.37; SD = 0.61; .sig02 = 0.00, 1.16;
Residual: Variance = 4.48; SD = 2.12; .sigma = 1.91, 2.27;
Number of obs.: 369, Care units: 115; Nursing homes: 64

Table S4 Mixed linear regression model for dimension *social relations* as dependent variable, the severity of each NPI-Q item defining the construct of agitation and the matching variables as fixed factors and care units nested in nursing homes as random factor

Independent Variables (fixed effects)	Coefficients	95% CI	SE	t	p
**(Intercept)**	**6.37**	**4.48, 8.27**	**0.98**	**6.50**	**<0.001**
**NPI-Q Severe Agitation/Aggression (yes)**	**-1.02**	**-1.51, -0.53**	**0.25**	**-4.03**	**<0.001**
NPI-Q Severe Disinhibition (yes)	0.30	-0.36, 0.96	0.34	0.87	0.384
**NPI-Q Severe Irritability/Lability (yes)**	**-1.12**	**-1.68, -0.56**	**0.29**	**-3.87**	**<0.001**
Age	0.01	-0.02, 0.03	0.01	0.59	0.558
Sex (female)	0.25	-0.17, 0.66	0.22	1.15	0.249
Visits (no)	-0.68	-1.48, 0.12	0.41	-1.65	0.101
DSS score	<0.01	-0.07, 0.07	0.04	0.04	0.970
Duration of stay	0.01	<-0.01, 0.01	<0.01	1.40	0.164
NPI-Q Delusions (no)	-0.13	-0.54, 0.28	0.21	-0.63	0.529
**NPI-Q Anxiety (no)**	**-0.61**	**-1.01, -0.21**	**0.21**	**-2.93**	**0.004**
NPI-Q Aberrant Motor (no)	-0.59	-1.02, -0.14	0.23	-2.60	0.010
NPI-Q Hallucinations (no)	0.64	0.19, 1.10	0.24	2.72	0.007

Random effects: Care unit (intercept): Variance = 0.10; SD = 0.32; .sig01 = 0.00, 0.93;
Nursing home (intercept): Variance = 0.36; SD = 0.60; .sig02 = 0.00, 0.96;
Residual: Variance = 2.98; SD = 1.73; .sigma = 1.56, 1.85;
Number of obs.: 369, Care units: 115; Nursing homes: 64

Table S5 Mixed linear regression model for dimension *social isolation* as dependent variable, the severity of each NPI-Q item defining the construct of agitation and the matching variables as fixed factors and care units nested in nursing homes as random factor

Independent Variables (fixed effects)	Coefficients	95% CI	SE	t	p
**(Intercept)**	**7.72**	**5.44, 10.01**	**1.18**	**6.54**	**<0.001**
**NPI-Q Severe Agitation/Aggression (yes)**	**-1.47**	**-2.06, -0.88**	**0.30**	**-4.84**	**<0.001**
**NPI-Q Severe Disinhibition (yes)**	**-1.52**	**-2.32, -0.72**	**0.41**	**-3.69**	**<0.001**
**NPI-Q Severe Irritability/Lability (yes)**	**-1.10**	**-1.78, -0.43**	**0.35**	**-3.16**	**0.002**
Age	-0.03	-0.05, <0.01	0.01	-1.85	0.065
Sex (female)	0.13	-0.37, 0.63	0.26	0.51	0.612
Visits (no)	0.25	-0.72, 1.21	0.50	0.50	0.619
DSS score	-0.04	-0.12, 0.05	0.04	-0.89	0.374
Duration of stay	<0.01	-0.01. 0.01	<0.01	0.04	0.968
NPI-Q Delusions (no)	0.29	-0.20, 0.78	0.25	1.14	0.253
NPI-Q Anxiety (no)	0.71	0.22, 1.20	0.25	2.83	0.005
NPI-Q Aberrant Motor (no)	0.35	-0.17, 0.87	0.27	1.29	0.197
NPI-Q Hallucinations (no)	0.40	-0.15, 0.95	0.29	1.41	0.161

Random effects: Care unit (intercept): Variance = 0.20; SD = 0.45; .sig01 = 0.00, 1.07;
Nursing home (intercept): Variance = 0.57; SD = 0.75; .sig02 = 0.00, 1.16;
Residual: Variance = 4.27; SD = 2.07; .sigma = 1.87, 2.21;
Number of obs.: 369, Care units: 115; Nursing homes: 64
